# Two cases of immune checkpoint inhibitor-associated diabetes mellitus induced by serplulimab: Case reports

**DOI:** 10.1097/MD.0000000000045414

**Published:** 2025-10-24

**Authors:** Yuan Gao, Meiyuan Lin, Jun Deng

**Affiliations:** a Department of Respiratory and Critical Care Medicine, The Affiliated Hospital of Southwest Medical University, Luzhou, Sichuan, China.

**Keywords:** immune checkpoint inhibitor-induced diabetes mellitus, immune-related adverse events, serplulimab, small cell lung cancer

## Abstract

**Rationale:**

Immune checkpoint inhibitors have shown promising efficacy in treating various malignancies; however, their use is associated with immune-related adverse events, including rare but severe immune checkpoint inhibitor–induced diabetes mellitus (ICI-DM). Serplulimab-induced ICI-DM has been rarely reported, and its clinical presentation can be heterogeneous.

**Patient concerns:**

We report 2 patients with small cell lung cancer who developed serplulimab-induced ICI-DM, both complicated by diabetic ketoacidosis (DKA). Case 1 developed delayed-onset ICI-DM after 2 years of treatment, with elevated glycated hemoglobin and moderate C-peptide deficiency. Case 2 developed ICI-DM earlier, with severe insulin deficiency and classic type 1 diabetes features. Neither patient tested positive for diabetes-related autoantibodies.

**Diagnoses:**

Both patients were diagnosed with serplulimab-induced ICI-DM, confirmed by hyperglycemia, C-peptide deficiency, and DKA during ongoing programmed cell death protein 1 inhibitor therapy.

**Interventions:**

Standard DKA management was administered for both patients. They were transitioned to subcutaneous insulin therapy and continued serplulimab treatment under close glucose monitoring.

**Outcomes:**

Both patients’ DKA was successfully controlled. They continued serplulimab and insulin therapy after discharge, with no recurrence of acute DKA.

**Lessons:**

These cases highlight the clinical heterogeneity and diagnostic challenges of ICI-DM. Routine blood glucose and glycated hemoglobin monitoring before and during immunotherapy may aid early detection. Clinicians should maintain a high index of suspicion for ICI-DM, even in patients without typical risk factors. Early recognition and insulin initiation are essential to prevent life-threatening complications of DKA. This report contributes to the limited literature on serplulimab-induced ICI-DM and provides practical insights into its diagnosis and management.

## 1. Introduction

Immunotherapy can modulate immune function and promote immune responses in cancer cells. Compared to traditional chemotherapy, it offers higher safety and better efficacy and has been widely used in the treatment of small cell lung cancer (SCLC).^[1]^ Although immune checkpoint inhibitors (ICIs) have shown significant therapeutic effects, they can induce autoimmune complications, known as immune-related adverse events (irAEs). Immune checkpoint inhibitor-induced diabetes mellitus (ICI-DM) is a rare irAE that typically presents with acute onset and often requires lifelong insulin therapy.^[2]^ Serplulimab, a programmed cell death protein 1 (PD-1) inhibitor, has been approved for the treatment of various cancers, including SCLC and non-small cell lung cancer. In this report, we present 2 cases of diabetes induced by serplulimab, both accompanied by diabetic ketoacidosis (DKA), with the aim of providing insights into the diagnosis and management of irAEs and raising awareness among clinicians.

## 2. Case report

### 2.1. Case 1

**Figure 1.**
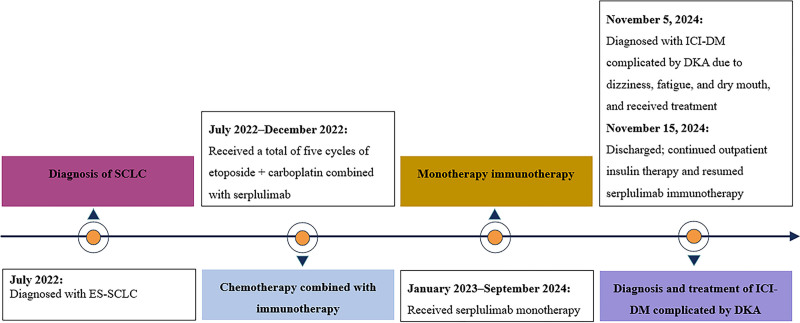
Case 1 – timeline of disease progression.

The patient was a 70-year-old man with a long history of smoking and alcohol consumption but no personal or family history of diabetes. In July 2022, he presented with respiratory symptoms, including cough and sputum production, at the Affiliated Hospital of Southwest Medical University. Chest computed tomography (CT) revealed central lung cancer in the right lung with possible obstructive pneumonia and atelectasis. Further fiberoptic bronchoscopy and pathological biopsy confirmed extensive-stage small cell lung cancer (ES-SCLC). On July 25, 2022, the patient received the first dose of serplulimab immunotherapy combined with etoposide and carboplatin. He underwent 5 cycles of this regimen until December 2022, after which he transitioned to serplulimab monotherapy. Throughout the treatment period, blood glucose levels were not significantly elevated. On November 5, 2024, the patient presented to our hospital with complaints of dizziness, fatigue, and dry mouth for more than one month, along with typical symptoms of diabetes, including polydipsia and polyuria. Upon admission, the vital signs were as follows: body temperature, 36.5°C; blood pressure, 133/82 mm Hg; heart rate, 102 bpm; respiratory rate, 24 breaths/min; oxygen saturation, 97%; height, 172 cm; and weight, 82 kg. Arterial blood gas analysis revealed primary metabolic acidosis, with a pH of 7.260 (normal range: 7.35–7.45), bicarbonate level of 5.21 mmol/L (normal range: 21.4–27.3 mmol/L), lactate level of 1.33 mmol/L (normal range: 0.50–1.60 mmol/L), blood ketone level of 7.3 mmol/L (normal range: 0.03–0.5 mmol/L), and blood glucose level of 27.2 mmol/L (normal range: 3.8–5.8 mmol). We suspected DKA. Further tests revealed glycated hemoglobin (HbA1c) of 11.9% (normal: 4–6%), fasting C-peptide level of 0.33 ng/mL (normal: 0.69–2.45 ng/mL), postprandial C-peptide level of 0.42 ng/mL (normal: 2.70–10.50 ng/mL), FT3 level of 0.36 pg/mL (normal: 1.80–3.80 pg/mL), FT4 level below the detectable minimum (<0.10 ng/dL, normal: 0.78–1.86 ng/dL), and TSH level above the detectable maximum (>100 mIU/L, normal: 0.38–5.57 mIU/L). Additionally, the pancreatic amylase level was 54.6 U/L (normal: 0–53 U/L) and the lipase level was 164.2 U/L (normal: 0–60 U/L). Autoantibodies associated with type 1 diabetes (T1D), including glutamic acid decarboxylase (GAD), protein tyrosine phosphatase, zinc transporter 8, and insulin antibodies, were negative. Upon admission, the patient was treated with the standard DKA protocol, including intravenous and continuous insulin infusion along with oral levothyroxine sodium. Given the patient’s medical history, we diagnosed ICI-DM and discontinued serplulimab immunotherapy. After blood glucose and ketone levels were controlled, the patient was switched to a subcutaneous insulin regimen with insulin aspartate. The patient was discharged after 10 days of treatment and continued insulin therapy and resumed serplulimab immunotherapy. The patient’s disease progression is shown in Figure 1.

### 2.2. Case 2

**Figure 2.**
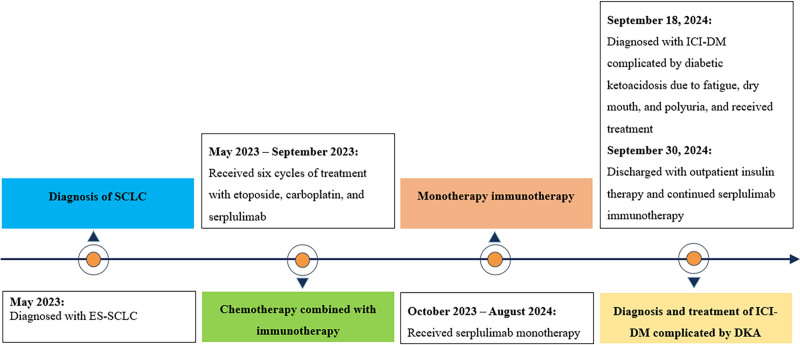
Case 2 – timeline of disease progression.

The patient was a 64-year-old man with a history of colon polypectomy, long-term smoking, and alcohol use but no personal or family history of diabetes. In May 2023, he presented to the affiliated hospital of Southwest Medical University for a chest CT scan, which revealed central lung cancer in the left lower lobe with possible obstructive pneumonia. Further fiberoptic bronchoscopy and pathological biopsy confirmed ES-SCLC. On May 19, 2023, the patient received the first dose of serplulimab immunotherapy combined with etoposide and carboplatin. He underwent 6 cycles of this regimen by September 2023, during which his blood glucose levels remained stable, with no significant increase. The patient then began regular monotherapy with serplulimab. On September 18, 2024, the patient presented to our hospital with complaints of fatigue, dry mouth, and polyuria for more than half a month. Upon admission, the patient appeared slightly lethargic and was afebrile. His vital signs were as follows: body temperature, 36.9°C; blood pressure, 102/61 mm Hg; heart rate, 83 bpm; respiratory rate, 20 breaths/min; oxygen saturation, 97%; height, 152 cm; and weight, 42.5 kg. Arterial blood gas analysis revealed primary metabolic acidosis, with a pH of 7.162 (normal: 7.35–7.45), bicarbonate level of 5.75 mmol/L (normal: 21.4–27.3 mmol/L), lactate level of 2.74 mmol/L (normal: 0.50–1.60 mmol/L), blood ketone level of 7.8 mmol/L (normal: 0.03–0.5 mmol/L), and blood glucose greater than the maximum detectable level of 33 mmol/L (normal: 3.8–5.8 mmol/L). We suspected DKA. Additional lab tests revealed HbA1c of 9.6% (normal: 4–6%), fasting C-peptide level of 0.03 ng/mL (normal: 0.69–2.45 ng/mL), postprandial C-peptide level of 0.05 ng/mL (normal: 2.70–10.50 ng/mL), insulin-like growth factor-1 level of 43.40 ng/mL (normal: 60.00–350.00 ng/mL), FT3 level of 0.9 pg/mL (normal: 1.80–3.80 pg/mL), FT4 level of 0.73 ng/dL (normal: 0.78–1.86 ng/dL), amylase level of 162.5 U/L (normal: 35–135 U/L), and lipase level of 73.3 U/L (normal: 0–60 U/L). Autoantibodies related to T1D, including GAD, protein tyrosine phosphatase, zinc transporter 8, and insulin, were negative. After admission, the patient was treated using the standard DKA protocol, including intravenous and continuous insulin infusion fluid resuscitation and oral levothyroxine sodium. Given the patient’s medical history, we diagnosed ICI-DM and discontinued serplulimab immunotherapy. After the blood glucose and ketone levels were controlled, the patient was switched to a subcutaneous insulin regimen with insulin aspartate. The patient was discharged after 11 days of hospitalization. Following discharge, the patient continued insulin therapy with good blood glucose control and resumed serplulimab immunotherapy with oral levothyroxine sodium. The disease progression is shown in Figure 2.

## 3. Discussion

**Figure 3.**
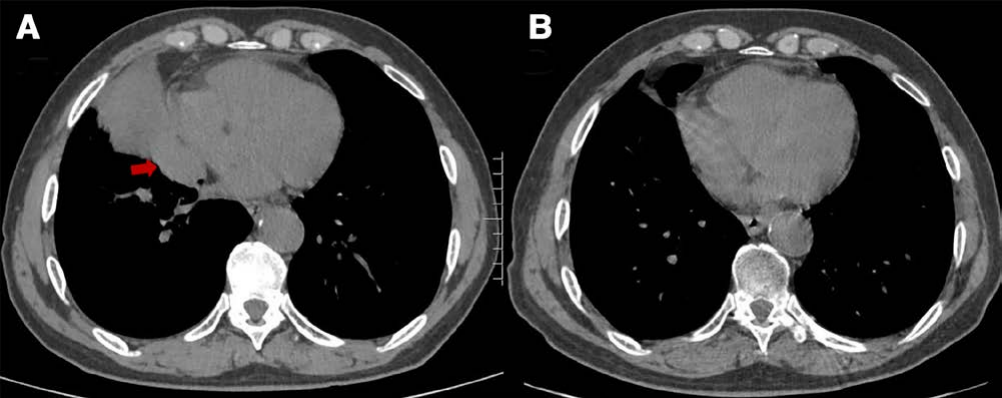
(A) CT scan on July 2, 2022, showing a mass of soft tissue in the middle lobe of the right lung (indicated by the red arrow) with bronchial obstruction and atelectasis. (B) Follow-up CT scan on November 6, 2024, demonstrating significant shrinkage of the right middle lobe mass after treatment, consistent with favorable therapeutic response and accompanied by marked relief of cough and sputum symptoms. CT = computed tomography.

**Figure 4.**
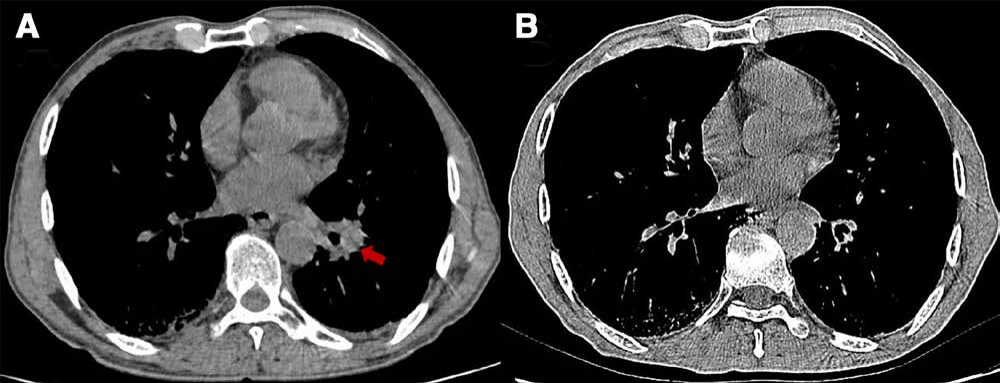
(A) CT scan on June 9, 2023, showing multiple nodules in the lower lobe of the left lung, the largest of which was approximately 3 cm in diameter, with spiculated edges (indicated by the red arrow). (B) CT scan on November 13, 2024, showing significant shrinkage of the nodules in the lower lobe of the left lung after treatment, with marked improvement in the patient’s cough and dyspnea. CT = computed tomography.

Immune checkpoints, including cytotoxic T-lymphocyte antigen 4 (CTLA-4), PD-1, and programmed death-ligand 1 (PD-L1), play critical roles in modulating immune responses. These molecules regulate T-cell proliferation and differentiation to enhance antitumor immunity and serve as key mediators of tumor immune evasion.^[2]^ Since the approval of ipilimumab, the first anti-CTLA-4 monoclonal antibody, in 2011,^[3]^ an increasing number of ICIs have been clinically approved, revolutionizing cancer treatment and significantly improving patient outcomes. Serplulimab, a fully humanized anti-PD-1 IgG4 monoclonal antibody, was approved by China’s National Medical Products Administration in March 2022 for the treatment of unresectable or metastatic MSI-H solid tumors in adults. In February 2025, it became the first PD-1 inhibitor approved by the European Union for ES-SCLC therapy.^[4,5]^ Clinical studies by Wang et al and Zhang et al demonstrated that serplulimab combined with chemotherapy outperformed previously established therapies (e.g., Atezolizumab, Durvalumab, and Pembrolizumab) in terms of both survival benefits and safety profiles in patients with ES-SCLC.^[6,7]^ In the present cases of 2 patients with SCLC, post-treatment chest CT scans demonstrated significant tumor regression (Figs. 3 and 4), with marked improvement in symptoms and enhanced quality of life, further confirming the efficacy of serplulimab.

However, the remarkable therapeutic potential of ICIs is associated with irAEs. ICIs disrupt inhibitory pathways to activate antitumor immunity; however, they may inadvertently trigger immune-mediated attacks on healthy tissues, leading to inflammatory conditions such as pneumonitis, colitis, hepatitis, and endocrine disorders (e.g., thyroiditis, hypophysitis, and diabetes). Notably, the incidence of ICI-DM ranges from 0.4 to 2%.^[2,8–10]^

The pathogenesis of ICI-DM remains unclear, although it predominantly occurs in patients receiving PD-1/PD-L1 inhibitors, with the highest incidence observed in combination therapies, such as PD-1 plus CTLA-4 blockade.^[10]^ PD-1, expressed on T lymphocytes, macrophages, natural killer cells, and dendritic cells, mediates immunosuppression by binding to its ligands PD-L1 (B7-H1/CD274) and PD-L2 (B7-DC/CD273). Notably, PD-L1 exhibits broad cellular expression patterns, including in pancreatic β-cells and malignant cells.^[11]^ Experimental evidence indicates that PD-L1-deficient mice develop accelerated non-obese diabetes, whereas PD-L1 overexpression protects β-cells from T cell-mediated attack, strongly suggesting a protective role against diabetic pathogenesis. PD-1/PD-L1 inhibitors may disrupt this balance through multiple mechanisms, ultimately leading to CD8^+^ T cell infiltration into the pancreatic islets and β-cell destruction.^[12,13]^ In contrast, the absence of CTLA-4 expression in pancreatic β-cells may explain the reduced association of CTLA-4 inhibitors with ICI-DM compared to that of PD-1/PD-L1-targeted therapies.

Currently, there are no standardized diagnostic criteria for ICI-DM. Diagnosis primarily relies on a history of ICI exposure and the exclusion of alternative causes of diabetes, with a median diagnostic timeframe of approximately 10 weeks.^[9,10,14]^ However, delayed-onset ICI-DM after treatment discontinuation has also been reported.^[15]^ Diabetes-associated autoantibodies are detectable in 30 to 50% of patients with ICI-DM, with GADA positivity being the most prevalent.^[16]^ Studies by Usui and Zhou et al suggested that patients positive for these autoantibodies progress to ICI-DM more rapidly.^[10,17]^ This pattern is consistent with our 2 reported cases, both of which exhibited negative autoantibody profiles and prolonged latency periods. In contrast, Marsiglio et al found no such correlation,^[18]^ which may be attributable to the limited sample size. Emerging evidence has linked specific human leukocyte antigen (HLA) haplotypes (e.g., DR4-DQ4 and DR9-DQ9) to ICI-DM susceptibility, with approximately 50% of patients testing positive for DR4-DQ4.^[16,19,20]^ In our cases, both patients developed ICI-DM over one year after starting treatment with serplulimab, with case 1 exhibiting symptom onset after 2 years of treatment. Although comprehensive testing for diabetes-associated autoantibodies yielded negative results, HLA class II genotyping was not performed on the patient. Neither HLA class II haplotypes nor diabetes-associated autoantibodies serve as diagnostic criteria, nor does their absence exclude ICI-DM diagnosis. Nevertheless, these biomarkers hold the potential for risk stratification, enabling clinicians to identify high-risk populations prior to immunotherapy initiation and to tailor therapeutic strategies accordingly.

Emerging reports have classified ICI-DM into 4 distinct subtypes. The first subtype, acute autoimmune T1D, is characterized by an abrupt onset and often presents with DKA. These patients typically exhibit normal or mildly elevated HbA1c levels, along with markedly reduced or undetectable C-peptide levels. The second subtype demonstrates slowly progressive hyperglycemia, resembling type 2 diabetes (T2D), with significantly elevated HbA1c levels but relatively preserved C-peptide levels. This form may arise from cytotoxic lymphocyte-mediated insulin resistance or tumor lysis-induced inflammation. The remaining subtypes include diabetes secondary to autoimmune pancreatitis and systemic lipodystrophy.^[18,21,22]^ In the 2 cases reported here, both patients presented with an acute onset of symptoms. Case 2 exhibited severe C-peptide depletion (0.1 ng/mL), confirming insulin-dependent ICI-DM. Case 1, however, displayed diagnostic ambiguity: markedly elevated HbA1c (11.9%) with only moderate C-peptide decline (0.6 ng/mL), absence of anemia or hemodialysis (factors known to distort HbA1c interpretation),^[23,24]^ and new-onset hyperglycemic symptoms (polyuria and polydipsia) within one month. Despite no personal or family history of diabetes, hypertension, dyslipidemia, or diabetes-associated autoantibodies, the patient’s overweight status (BMI 28.4) suggested concurrent insulin resistance alongside acute T1D pathogenesis. This is consistent with the findings of Hong et al,^[25]^ who described ICI-DM superimposed on preexisting T2D, and Bastin et al,^[26]^ who reported nivolumab-induced hyperosmolar hyperglycemia in a patient with a family history of T2D. Notably, while routine fasting glucose monitoring in Case 1 revealed no abnormalities, postprandial hyperglycemia, potentially indicative of insulin resistance, may have been overlooked because of inadequate post-meal glucose tracking. This underscores a critical clinical gap: although some studies suggest that DKA in ICI-DM occurs unpredictably,^[27]^ Marsiglio et al observed gradual glycemic elevation 3 weeks prior to T1D onset,^[18]^ highlighting the need for vigilant glucose monitoring to preempt severe complications. Additionally, approximately one-third of ICI-DM cases exhibit nonspecific elevations in amylase and lipase levels, indicating pancreatic exocrine dysfunction.^[28]^ Both patients demonstrated mild enzyme elevations without abdominal pain or CT evidence of pancreatitis, likely reflecting subclinical inflammatory effects on the pancreatic tissue.

Although ICI-DM is rare, it is largely irreversible and requires early recognition and intervention to mitigate diabetes-related complications. Before initiating ICIs therapy, baseline blood glucose and HbA1c levels should be established. During treatment, fasting and postprandial blood glucose levels should be monitored, and C-peptide and diabetes-related autoantibodies can be assessed when necessary. In patients receiving ICIs, attention should be paid to new-onset symptoms, such as polyuria, polydipsia, weight loss, and fatigue, and patients should be educated to recognize high-risk symptoms. Even after discontinuation of therapy, extended follow-up is recommended to detect delayed-onset ICI-DM.^[15]^ Current management predominantly relies on insulin therapy, whereas corticosteroids are contraindicated because they tend to exacerbate hyperglycemia without proven clinical benefits.^[1]^ For patients with mild symptoms and well-controlled blood glucose levels (≤8.9 mmol/L), ICIs treatment may cautiously continue under rigorous monitoring. However, severe hyperglycemia or complications necessitate ICIs discontinuation until glycemic stability is restored, after which ICIs reintroduction should be evaluated judiciously.^[29]^ Notably, 50 to 70% of patients present with DKA at diagnosis,^[16,30]^ as observed in both cases reported here. Acute management mandates prompt intravenous insulin infusion, fluid resuscitation, hourly glucose monitoring, and serial assessments of pH, electrolyte levels, and renal function. Following stabilization, patients typically transition to lifelong subcutaneous insulin regimens under endocrinological supervision, supplemented by regular follow-up and patient education on glucose monitoring and DKA prevention.^[1,31]^ Emerging evidence suggests an exploratory role for immunotherapy and regenerative strategies. A case report by Trinh et al demonstrated partial reversal of ICI-DM using rituximab,^[32]^ although mechanistic insights and broader applicability remain unconfirmed. Preclinical studies have highlighted the potential of mesenchymal stem cells to attenuate pancreatic islet infiltration by T-cells and CXCL9 + macrophages, reducing the incidence of ICI-DM in murine models;^[33]^ However, human trials are pending. Concurrent irAEs, particularly endocrine disorders such as hypothyroidism, adrenal insufficiency, and thyroid dysfunction, frequently accompany ICI-DM and require parallel management, as seen in both our patients. After the diagnosis of ICI-DM in both patients, we communicated the condition and its implications to the patients and their families. Patient 1 expressed distress over developing ICI-DM, whereas Patient 2 was more accepting. This difference resulted in lower adherence to insulin therapy during follow-up in Case 1 than in Case 2. Therefore, it is essential to provide detailed counseling on the potential adverse effects of ICIs before treatment and to maintain close supervision during the long-term management of patients diagnosed with ICI-DM. In summary, the cornerstone of ICI-DM management is lifelong insulin therapy combined with comprehensive patient education. Empowering patients to recognize early symptoms (e.g., polyuria, polydipsia, and weight loss) and adhere to glucose monitoring protocols is critical for averting severe complications, such as DKA.^[34]^ Although novel therapies hold promise, their clinical translation requires robust validation.

In conclusion, we present 2 cases of serplulimab-induced ICI-DM, with only one previously reported instance of serplulimab-related ICI-DM identified in the literature to date.^[35]^ Notably, although both patients received the same therapeutic agent, their clinical manifestations differed significantly, underscoring the phenotypic heterogeneity of this adverse event. While the underlying mechanisms remain incompletely elucidated, these cases highlight the need for vigilant monitoring of glucose metabolism parameters, including fasting blood glucose and HbA1c, before, during, and even after discontinuation of immune checkpoint inhibitor therapy to detect both acute and delayed-onset ICI-DM. Furthermore, the management of ICI-DM warrants increased clinical attention and expanded research efforts to establish standardized therapeutic protocols and identify predictive biomarkers.

## Acknowledgments

We would like to express our sincere gratitude to the patients for granting permission to use their clinical data for this study and for their invaluable contributions to the publication of this research.

## Author contributions

**Conceptualization:** Yuan Gao.

**Data curation:** Meiyuan Lin.

**Writing – original draft:** Yuan Gao.

**Writing – review & editing:** Jun Deng.
